# Prevalence, vector density and associated risk factors of caprine trypanosomosis: a cross-sectional study in Arbaminch, Southern Ethiopia

**DOI:** 10.1016/j.vas.2026.100668

**Published:** 2026-05-01

**Authors:** Hirut Getnet Tegegn, Assaye Wollelie Fentie, Animaw Andargie Worku, Assaye Desta Amare, Teketay Bayleyegn Derso, Yihenew Getahun Ambaw, Getachew Tadesse Siyoum

**Affiliations:** Department of Veterinary Medicine, College of Agricultural Sciences, Woldia University, Woldia, Ethiopia

**Keywords:** Caprine, Ethiopia, Prevalence, Risk factors, Trypanosomosis, Vector density

## Abstract

Caprine trypanosomosis remains a neglected health constraint to goat production in Ethiopia. Despite studies conducted on caprine trypanosomosis in other areas of the country, limited information exists in and around Arbaminch area. This study aimed to investigate the prevalence, vector density and risk factors of caprine trypanosomosis in and around Arbaminch town, Southern Ethiopia. A cross-sectional study design was employed from December 2024 to April 2025. Blood samples were collected from 384 randomly selected goats and examined using hematological and parasitological techniques, and entomological surveys were performed with 26 traps deployed across three sites. Data were summarized using descriptive statistics and chi-square tests were used to assess associations of infection with risk factors. The overall prevalence of caprine trypanosomosis was 7.55%, with *Trypanosoma vivax* (4.95%) and *Trypanosoma congolense* (3.65%) recorded as the causative species. Poor body condition, adult age, and black coat color were significantly (*p* < 0.05) associated with infection, while sex was not. Mean packed cell volume was lower in parasitaemic goats (20.52%) compared to aparasitaemic goats (28.61%). Entomological surveys identified *Glossina pallidipes* as the only tsetse species, with an apparent density of 8.3 flies per trap per day (FTD), alongside *Stomoxys* and *Tabanus* species. This finding indicates that caprine trypanosomosis constitutes a relevant health concern in Arbaminch, particularly when considered alongside the high density of tsetse vectors. Although the prevalence is moderate, it highlights the need for continued surveillance and targeted interventions to safeguard goat health and productivity in the region.

## 1 Introduction

Ethiopia is believed to have the largest livestock population in Africa and the fifth largest in the world, with over 66 million cattle, 38 million sheep, 46 million goats, 56 million chickens, 2 million horses, 10 million donkeys, 360,000 mules, and 7 million camels (CSA, 2021/22). Among these, goats play a particularly important role in the livelihoods of smallholder farmers, serving as a source of meat, milk, income, and cultural value. Their adaptability to diverse agro-ecological zones makes them a critical resource for both subsistence and economic development (Endalew & Ayalew, 2016). Despite this importance, goat productivity remains low, largely due to disease constraints. One of the most significant diseases affecting goats is trypanosomosis, which severely hampers health, reproduction, and productivity (Afework, 2006). The disease imposes direct losses (e.g., reduced milk yield, infertility, and abortion) and indirect costs (e.g., replacement expenses, reduced market value), collectively undermining household income and food security (Irungu et al., 2002; Abro et al., 2021; Adeyemo et al., 2022; Ali et al., 2019).

Caprine trypanosomosis is caused by protozoan parasites of the genus *Trypanosoma,* family *Trypanosomatidae* (FAO, 2025; Oche & Kasahun, 2025; WOAH, 2024). In Ethiopia, the most important species affecting goats include *T. brucei, T. congolense*, and *T. vivax*, which are of significant importance in African animal trypanosomosis (Getachew, 2005). Disease transmission occurs primarily through tsetse flies (*Glossina* spp.), whose distribution is strongly influenced by vegetation, humidity, and temperature (Krinsky, 2019; Shiferaw, 2021). These ecological factors contribute to the persistence of the disease in endemic areas. Recent national tsetse belt mapping studies indicate that Ethiopia’s agriculturally fertile lands heavily infested, covering approximately 150,000 km² across the western and southwestern regions (Gebre et al., 2022). Five *Glossina* species are documented: *G. morsitans submorsitans, G. pallidipes, G. fuscipes fuscipes, G. tachinoides*, and *G. longipennis*. Among these, the first four continue to pose major medical and economic importance, while *G. longipennis* is of minor relevance (Shiferaw *et al*., 2021; Gebre et al., 2022). Other biting flies such as *Tabanus* spp. can also serve as mechanical vectors, particularly in non-tsetse areas (Delano et al., 2007). The epidemiology of caprine trypanosomosis is influenced by several factors, including, environmental conditions, host susceptibility and the presence of vectors (Fetene et al., 2021). Importantly, vector abundance and disease incidence often follow seasonal patterns, with peaks during rainy periods when environmental conditions favor fly populations (Kasozi et al., 2019).

The control of trypanosomosis relies on integrated approaches targeting both vectors and host management. Vector control strategies include the application of insecticide-treated nets and sprays, which have been shown to reduce exposure of goats to tsetse flies and other biting insects (Ayaz et al., 2018; Sargison, 2020). Rotational use of insecticides is recommended to mitigate the development of resistance among vector populations (Dossa, 2007). In addition, baited traps deployed in grazing areas have demonstrated effectiveness in reducing local fly densities by attracting and capturing tsetse flies (Abebe et al., 2017)). Environmental management practices, such as clearing bushy vegetation and maintaining open grazing spaces, contribute to the reduction of suitable habitats for tsetse flies (Ayaz et al., 2018). From the host perspective, routine health monitoring facilitates early detection of trypanosomosis, while quarantine of newly acquired animals reduces the risk of introducing infection into herds. Adequate nutrition further enhances the resilience of goats against parasitic infections (Dossa, 2007).

Although numerous studies have investigated caprine trypanosomosis in Ethiopia, there is limited information specific to the Arbaminch area. In particular, the density and distribution of tsetse and other biting flies in relation to caprine trypanosomosis remain poorly characterized. This represents a critical knowledge gap, as Arbaminch is an area where goat production is economically and socially important, yet burden of caprine trypanosomosis is not well documented. Addressing this gap is vital for designing effective, locally adapted control strategies. The general objective of this study is to assess the epidemiology of caprine trypanosomosis in Arbaminch, Southern Ethiopia, with particular emphasis on prevalence, vector density, and associated risk factors. To achieve this, the study pursues three specific objectives: (1) to determine the prevalence of caprine trypanosomosis in Arbaminch and its surrounding areas, (2) to estimate the density and distribution of tsetse and other biting flies in relation to goat production systems, and (3) to identify associated related risk factors that contribute to disease occurrence. Thus, integrating entomological data with epidemiological surveys is essential to fully understand disease dynamics in this region.

## 2 Materials and methods

### 2.1 Description of the study area

The study was conducted in and around Arbaminch town (Fig. 1 from December 2024 to April 2025. Arbaminch is located in the Southern Rift Valley of Ethiopia, lying between latitude 5°57′N and longitude 37°32′E. Arbaminch lies within the Gamo Zone, approximately 500 km south of Addis Ababa and 250 km southwest of Hawassa. The climate alternates with long summer rainfall (june-september), while a shorter rainy season from march to may, and winter dry season (december-february) with mean annual rain fall of 750 to 930  mm. The mean annual temperature is 30°C which is between 10°C and 37°C and the altitude ranges from 1200 to 3125  m above sea level. The vegetation is predominantly wood-grassland, especially along grazing areas and drainage lines. This rainfall pattern, combined with temperature and vegetation pattern, provides favorable ecological conditions for tsetse fly survival and transmission (Seyoum et al., 2022).

**Fig. 1:**
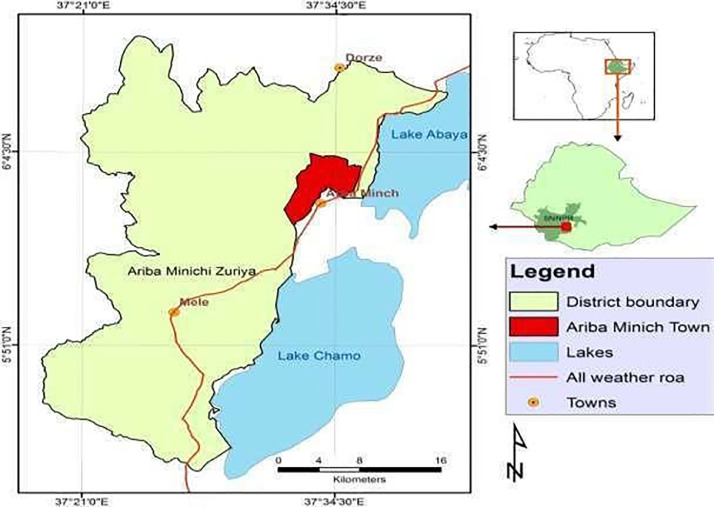
Map showing the study area (Source: Alemu & Desta, 2017).

The name Arbaminch also means “springs”. The town houses the uptown administrative center of Secha which is 4 km away from the downtown commercial and residential areas of Sikela. On the Eastern part, Sikela bounds with Nechisar National Park, Lake Abaya to the North and Lake Chamo to the South. Also, Kulfo River flows along the center of the town and drains into Lake Chamo. The system of agriculture practiced in the area is mixed livestock farming system (Gamo Goffa Zone Agricultural, & Rural Development office GZARDO, 2007).

### 2.2 Study animals

The study animals were local breed goats, which were kept under extensive management system in different kebeles in and around Arbaminch town. The goats included in this study were of different age groups, both sex, different colors and body condition.

### 2.3 Study design

A cross-sectional study design was adopted for the study to assess the prevalence, vector density and risk factors of caprine trypanosomosis in and around Arbaminch town, southern Ethiopia. Samples were collected between December 2024 and April 2025.

### 2.4 Sample size determination and sampling technique

The desired sample size for the study was determined using the formula described by (Thrusfield, 2007), assuming an expected prevalence of 50%. This conservative estimate was chosen because no prior data on caprine trypanosomosis in Arbaminch was available, and 50% maximizes sample size to ensure adequate statistical power.

$$n\;=\frac{{\left(1.96\right)}^{2}\times P\left(1-P\right)}{d^{2}}$$

where, n= the required sample size; 1.962= the value of Z, at confidences 95% level; P=expected prevalence; d= desired absolute precision level. Accordingly, the calculated sample size was 384 goats. Multistage sampling was employed to select the study area and animals. In the first stage, Arbaminch was selected purposively based on accessibility, potential goat population, and absence of prior study. Thereafter, three sites namely Abulo airport area, Kola Shara and Crocodile ranch were selected randomly from the other areas. Finally, 384 households owning goats were randomly selected, after which one goat was randomly selected from each household for sampling.

### 2.5 Data collection protocols

#### 2.5.1 Parasitological and hematological examination

Diagnosis of caprine trypanosomosis was based on parasitological methods, specifically the wet smear and buffy coat technique (BCT). Microscopy was chosen for this study due to its simplicity, cost‑effectiveness, and applicability under field conditions in endemic areas. From each animal, blood samples were collected in a pair of heparinized capillary tubes by puncturing the ear veins using sterile blood lancet. The tubes were filled to three-quarters of their capacity and sealed at one end using crystal seal. Then the capillary tubes were loaded on the micro hematocrit centrifuge symmetrically and centrifuged at 12,000 rpm for 5 min (OIE, 2018).

Packed cell volume (PCV) was determined using a hematocrit reader. After PCV determination, the capillary tubes were broken 1 mm below the buffy coat layer using a diamond pencil to include the red blood cell layer. The contents were expressed onto a microscopic slide, covered with a 22 × 22 mm cover slip, and examined under a 40 × objective lens using the dark‑ground buffy coat technique (Desquesnes et al., 2022).

In addition, wet smear were prepared and examined for motile trypanosomes in all animals tested (OIE, 2017). From BCT‑positive samples, thin blood smears were prepared, fixed with methanol for 5 min, stained with Giemsa solution for 30 min, and examined under oil immersion at 100 × objective to identify trypanosome species (Desquesnes, 2017; Woo, 1996). Species identification relied on morphological features such as size, position of the kinetoplast, development of undulating membranes, and presence of a free flagellum.

#### 2.5.2 Entomological survey

A total of 26 standard Nguruman (NGU) traps were deployed in Abulo airport area, kola Shara and Corocodile ranch in different time at the month of February. NGU traps were constructed from locally available materials in order to attract and catch the flies. All traps were uniformly baited with acetone and cow urine. The traps were deployed at an interval of 200 m apart (FAO, 1992). Traps were deployed for a period of 72 h before collection. Trap deployment sites were selected to represent all habitat or vegetation types in the study area that could be associated with tsetse fly feeding, behavior, reproduction, and other related aspects. The poles of each trap were carefully greased to prevent tsetse fly predators mainly ants. Then, tsetse and other biting flies trapped were collected and counted (FAO, 1992; Tora et al., 2022). They were sorted by sex and species. Identification of the tsetse and other biting flies collected was done by characteristic morphological features such as size, color, proboscis and wing venation (Tadesse et al., 2010). Sexing was also done for the flies just by observing the posterior end of the ventral aspect of abdomen by hand lens. Male flies are easily identified by enlarged hyphophagym. The number of flies caught per trap per day was determined to calculate the flies’ density (FAO, 1992; Utino et al., 2024).

### 2.6 Data management and analysis

The collected data was stored, managed and coded in Microsoft excel spread sheet and thereafter exported to SPSS version 20 for statistical analysis. Tables and graph were used for data presentation. The prevalence of infection was computed by dividing the number of infected samples by the total number of sampled multiplied by 100. The difference in mean PCV values between Parasitaemic and aparasitaemic goats were compared using independent *t*-test. The association of trypanosomosis (dependent variable) with different independent variables (age, sex, body condition score (BCS), and coat color) was assessed using multivariate logistic regression analysis. In univariable logistic regression analysis, predictors with *P* value (p < 0.25) were subjected to multicolinearity assessment and considered for multivariable logistic regression analysis. The final model was developed using inter-method multivariable logistic regression analysis. Model fit was checked in Hosmer and Lemeshow (Dohoo et al., 2009). The association was statistically significant if *p*-value ˂ 0.05. The apparent density of tsetse and biting flies was expressed as the number of flies per traps per day (FTD), which is a continuous variable (Abera, 2022).

## 3 Results

### 3.1 Prevalence and risk factors of caprine trypanosomosis

The overall prevalence of caprine trypanosomosis in the study area was 7.55% (29/384). Specifically, prevalence by BCT was 7.55% (29/384), while prevalence by wet smear was 3.65% (14/384), reflecting the lower sensitivity of wet smears under low parasitemia conditions. The prevalence of *T. vivax, T. congolense* and co-infection (both *T. vivax* and *T. congolense*) was 4.95%, 3.65% and 1.04%, respectively (as shown in Table 1).

**Table 1:** Prevalence of caprine trypanosomosis by species in the study area (n = 384).

Species	Positive animals	Prevalence%
*T. vivax*	15	4.95
*T. congolense*	10	3.65
Co-infection	4	1.04
**Total**	**29**	**7.55**

Table 2 below presents the crude and adjusted odds ratios (cOR & aOR), 95% confidence intervals (CI), and *p*-values for each predictor variable. Multivariable logistic regression analysis was performed to assess the association of risk factors such as sex, age, body condition score, and coat color with the occurrence of caprine trypanosomosis. The risk factors considered in the univariable logistic regression analysis of the presence of trypanosomosis were age, sex, BCS, and coat color. All the factors were found to be significantly (*p*< 0.25) associated with trypanosomosis (Table 2). After checking for collinearity, all significant variables in the initial analysis were subjected to inter-method multivariable logistic regression analysis. Accordingly, all of the four variables retained as significant (*p*< 0.05) predictors of trypanosomosis in the final model. The Hosmer-Lemeshow goodness-of-fit test suggested that the model fit the data (χ²= 10.38; *p*= 0.2397) (Table 2). The overall model was statistically significant, indicating that these host-related factors collectively contributed to variation in disease status.

**Table 2:** Multivariate logistic regression analysis of risk factors for caprine trypanosomosis.

Variables	Categories	No examined	No positive (%)	cOR (95%CI)	*P*-value	aOR (95%CI)	*P*-value
Age	Young*	240	13(5.42)				
	Adult	112	13(11.61)	2.29(1.03–5.12)	0.043	2.46(1.00–6.02)	0.049
	Old	32	3(9.38)	1.81(0.49–6.72)	0.378	1.37(0.33–5.72)	0.664
Sex	Male*	188	10(5.32)				
	Female	196	19(9.69)	1.91(0.86–4.23)	0.110	2.14(0.89–5.16)	0.091
BSC	Good*	73	3(4.11)				
	Medium	223	7(3.14)	0.76(0.19–3.00)	0.691	0.65(0.16–2.64)	0.544
	Poor	88	19(21.59)	6.43(1.82–22.70)	0.004	5.29(1.46–19.17)	0.011
Color	white*	64	1(0.26)				
	Black	128	15(11.72)	8.36(1.08–64.80)	0.042	8.63(1.05–71.17)	0.045
	Mixed	125	9(7.20)	4.89(0.61–39.46)	0.136	5.11(0.59–43.94)	0.138
	Red brown	67	4(5.97)	4.00(0.43–36.79)	0.221	4.15(0.42–40.90)	0.223
	**Total**	**384**	**29(7.55)**				

⁎ Good body condition, young, male, and white coat color were used as reference groups. cOR; crude odds ratio, aOR; adjusted odds ratio, BSC; Body Condition Score.

There was no statistically significant difference between sexes (*P*= 0.091), although females showed a higher prevalence (9.69%) compared to males (5.32%). Age was significantly associated with infection, with adult goats having higher odds (*P*= 0.049) compared to young ones, while old goats did not differ significantly (*P*= 0.664). Body condition score showed a strong effect (*P*= 0.011), as goats in poor condition were more than five times as likely to be infected compared to those in good condition. Coat color was also significantly associated (*P*= 0.045), with black-coated goats exhibiting the highest prevalence (11.72%), followed by mixed (7.2%) and red-brown (6.8%), while white-coated goats had the lowest prevalence (0.26%).

#### 3.1.1 Packed cell volume (PCV) of parasitemic and aparasitemic goats

The mean PCV of aparasitaemic goats (28.61%) was significantly higher (*p* = 0.001, 95% CI: 28.00–29.21) compared to parasitaemic goats (20.52%, 95% CI: 19.69–21.34). Independent samples t‑test analysis confirmed that parasitaemic goats had markedly reduced PCV values (t = 7.47, df= 382, p < 0.001), indicating a strong association between trypanosome infection and anemia (Table 3).

**Table 3:** Comparison of mean PCV values between parasitaemic and aparasitaemic goats.

Infection status	Mean of PCV	95% CI	*t*-value	df	*p*-value	Total
Parasitaemic	20.52	19.69–21.34	7.47	382	˂0.001	29
Aparasitaemic	28.61	28.00–29.21				355
**Total**						**384**

### 3.2 Entomological survey of tsetse and other biting flies

A total of 26 NGU traps were used for trapping flies for a period of 72 h (Table 4). Overall, 857 flies were caught of which 645(75.26%) were *Glossina* species. Other biting flies encountered during the study period include 251(11.79%) *Stomoxys* species and 111(12.95%) *Tabanus* species. The overall apparent density of *Glossina* species was 8.3 F/T/D whereas that of the other biting flies was 2.7 F/T/D. Only one *Glossina* species (*G. pallidipes*), was identified in this study area.

**Table 4:** Entomological survey summary of tsetse and biting flies in Arbaminch.

Trap sites	No. of traps	Days	Tsetse flies		Other biting flies						
			*G. pallidipes*	F/T/D	*Stomoxys* Sp.	*Tabanus* Sp.		Total		F/T/D	
Crocodile ranch	9	3	294	10.9	48		57		105		3.9
Abulo airport	9	3	189	7	25		31		56		2.1
Kola shara	8	3	162	6.8	28		23		51		2.1
**Overall**	**26**	**3**	**645**	**8.3**	**101**		**111**		**212**		**2.7**

The distribution of the *Glossina* species across location and sex is contained in Table 5. Female *Glossina* Sp. Were more in number accounting for 65.58% (n = 423) of the collection compared to the male flies (34.42%, n = 222). The site with the most tsetse flies captured was Crocodile ranch (294), followed by Abulo airport area (189), and the least number of flies collected from Kola shara (162).

**Table 5:** Distribution of *Glossina pallidipes* based on sex.

Trap sites	No of traps	Sex		Total
		Female	Male	
Crocodile ranch	9	207(70.41%)	87(29.59%)	294
Abulo airport area	9	117(61.9%)	72(38.1%)	189
Kola shara	8	99(61.11)	63(38.89%)	162
**Overall**	**26**	**423(65.58%)**	**222 (34.42%)**	**645**

*F/T/D means flies collected per trap per day.

## 4 Discussion

This study demonstrates several notable strengths that enhance both its scientific rigor and practical relevance. By integrating parasitological, hematological, and entomological approaches, it offers a comprehensive perspective on trypanosome infections and their epidemiological context. The focus on goats which are host species often neglected in trypanosomosis research addresses a critical gap and draws attention to the role of neglected livestock in endemic areas such as the Arbaminch tsetse belt. The epidemiological sample size (n = 384) provides sufficient power to ensure reliability and representativeness of the findings. Moreover, the combined assessment of infection status with PCV analysis delivers clinically meaningful insights, linking parasitological data with indicators of animal health and productivity. Explicitly highlighting these strengths underscores the balanced contribution of the study and enhances its overall impact.

This study revealed that caprine trypanosomosis remains a relevant constraint to goat production in Arbaminch, with an overall prevalence of 7.55%, aligning with reports from Gabon (7.8%) (Maganga et al., 2020) but exceeding earlier Ethiopian estimates. For instance, the current finding is higher than the 3.56% reported in Didessa and Ghibe Valley (Dinka & Abebe, 2005), 4.1% in Guangua district (Kebede et al., 2009), 1.96% in Benishangul Gumuz (Lelisa et al., 2016), and 1.07% in Uganda (Rascon García et al., 2023). However, our findings are lower than those reported by other authors, ranging from 12–18% in Nigeria depending on the region (Abenga et al., 2020), 20.05% in Abrahamo Woreda (Aki, 2025), and 18.75% in Southern Punjab, Pakistan (Tariq et al., 2024). Such variation likely reflects ecological differences, season of the study period, vector challenge, control efforts, and farmer awareness. Importantly, it positions Arbaminch as a relatively higher prevalence compared to certain Ethiopian regions where goats face a heavier burden than in many other Ethiopian regions, warranting targeted interventions (Degneh et al., 2017; Amante & Tesgera, 2020).

Only two trypanosome species were encountered in this study with higher occurrence of *T. vivax* compared to *T. congolense*, consistent with findings from Western Ethiopia (Tadese & Megersa, 2010), but with higher proportions than those reported by Tamiru et al. (2021). These comparisons pointout Arbaminch as a tsetse-belt area where goats face heavier trypanosome challenge than in many other Ethiopian regions.

The multivariable logistic regression analysis identified body condition score, age, and coat color as significant predictors of caprine trypanosomosis. Goats with poor body condition were over five times more likely to be infected compared to those in good condition (OR= 5.29, p = 0.011), underscoring the role of nutritional and health status in susceptibility to parasitic diseases. This observed association between poor body condition and infection is consistent with previous reports (Lelisa et al., 2016; Tamiru et al., 2021; Oche & Kasahun, 2025). Age-related differences were also observed, with adult goats showing more than twice the odds of infection compared to young goats (OR= 2.46, *p*= 0.049), while old goats did not differ significantly. This may reflect increased exposure risk among adults or acquired immunity among older stock.

Coat color also emerged as an important factor, with black-coated goats exhibiting markedly higher odds of infection than white-coated goats (OR= 8.63, *p*= 0.045). This finding is in line with evidence that tsetse flies are strongly attracted to dark colors, a phenomenon confirmed in African vector control research (Santer, 2017) and supported by national mapping of tsetse distribution in Ethiopia (Gebre et al., 2022). Although female goats tended to have higher odds of infection than males (OR= 2.14), this association did not reach statistical significance (*p*= 0.091). Overall, these results highlight the importance of host-related factors in shaping trypanosomosis risk and suggest that management strategies should prioritize improving body condition, considering coat color in breeding or herd composition, and focusing preventive measures on younger animals.

The mean PCV of infected animals (20.52%) was significantly lower than that of non-infected animals (28.61%) (*p*< 0.05). The reduction in PCV among parasitaemic goats highlights the hematological impact of trypanosome infection, indicating the well‑established role of trypanosomes in inducing anemia through hemolysis, bone marrow suppression, and increased erythrophagocytosis. This finding is consistent with report of Tamiru et al. (2021); Oche and Kasahun (2025); Lelisa et al. (2016). Such reductions in PCV are likely to impair productivity through decreased milk yield, growth rates, and reproductive performance, thereby exacerbating household economic vulnerability. The mean PCV of infected goats (20.52%) fell below the threshold (24%) commonly used to define anemia in small ruminants (Radostits et al., 2007), underscoring the clinical relevance of this finding. Similar reductions in PCV have been reported in Ethiopian field studies, where trypanosome‑positive goats and sheep consistently exhibited lower hematocrit values compared to aparasitaemic counterparts (Tamiru et al., 2021).

In this study, the only *Glossina* species encountered was *G. pallidipes* with high apparent density (8.3 FTD), and females predominating the collection, suggesting sustained transmission potential. The presence of *Stomoxys* and *Tabanus* species highlights the risk of mechanical transmission, particularly during seasonal peaks. Although apparent density of *Glossina* species was lower than some previous studies (Tilahun et al., 1997), the predominance of female flies suggests sustained transmission potential. Seasonal variation was not captured due to the cross-sectional design, representing a limitation that future longitudinal studies should address. In the present entomological survey, a total of 645 (75.26%) tsetse flies and 212 (24.74) other biting flies (*Stomoxys* and *Tabanus*) were trapped. *G.pallidipes* was the only species of *Glossina* caught in the area. This observation is consistent with previous studies, which reported *G. pallidipes* as the single or predominant tsetse fly in Southwest areas and Omo-Ghibe tsetse belt of Ethiopia (Abebe et al., 2017). Moreover, the finding is in agreement with the report of previous studies conducted in different part of southern Ethiopia (Abera, 2022; Ayele et al., 2012; Girma et al., 2014; Sheferaw et al., 2016; Teka et al., 2012; Zeleke, 2011). The overall apparent densities of *G. pallidipes* and biting flies in the study area were 8.3 F/T/D and 2.7 F/T/D, respectively, which were lower than the previous reports by Rodrigues et al. (2019) and Abera (2022); Teka et al. (2012) with an overall apparent *G. pallidipes* densities of 29.624 F/T/D, 47.8 F/T/D and 8.3 F/T/D, respectively. These variations could be due to differences in seasons, vegetation type, availability of host animals and control strategies applied in the respective places (Cecchi et al., 2024; Leak et al., 1993; Nnko et al., 2017).

The density of female *G. pallidipes* was higher compared with male flies. This is similar to the results obtained by Lemu et al. (2019); Efa (2021); Eyasu et al. (2021); Abera (2022) in different parts of the country. This could suggest female flies physiologically necessitated to feed more animal blood during pregnancy than males which exposes it to trapping than male tsetse flies (Leak, 2009; Kassaye, 2015). Among the mechanical vectors found in present study area, *Tabanids* were slightly higher than *Stomoxys* in the Crocodile ranch and Abulo airport trapping sites. On the other hand, *stomoxys* were slightly higher than *Tabanids* Kola shara site. The presence of mechanical flies in the areas cannot be underestimated as they have been shown to mechanically transmit *Trypanosoma* (Desquesnes et al., 2009) and can be responsible for seasonal epidemic patterns in low tsetse density areas (Kone et al., 2011; Rahman, 2005).

While this study provides valuable baseline data on caprine trypanosomosis in Southern Ethiopia, several methodological constraints should be acknowledged. First, reliance on conventional microscopy may have limited diagnostic sensitivity, potentially underestimating true infection prevalence compared to molecular methods. Second, the cross‑sectional design restricts the ability to capture seasonal variation and temporal dynamics of infection. Third, the entomological survey was conducted over a relatively short duration, which may not fully represent vector abundance and distribution across different seasons. Finally, the sampling approach did not adjust for potential clustering effects, which could influence precision of prevalence estimates.

Future research should address these limitations by incorporating molecular diagnostics for species confirmation, adopting longitudinal designs to assess seasonal patterns, extending entomological monitoring, and applying multivariate analyses to account for management practices, drug use, and clustering effects. Such methodological improvements will enhance scientific rigor and generate more actionable insights to protect goat populations and strengthen rural livelihoods.

## 5 Conclusion and recommendations

This study revealed that the prevalence of caprine trypanosomosis in Arbaminch, Southern Ethiopia is7.55% demonstrating that goats face a significant disease burden from the infection. Infection with *T. vivax* was slightly higher compared with *T. congolense* even though, mixed infection was also observed. The body condition of the goats was associated with the prevalence of trypanosomosis, with marked reduction in packed cell volume among infected animals confirming the clinical impact of the disease, with implications for productivity and household livelihoods. Entomological findings identified *G. pallidipes* as the sole tsetse species in the area, with high apparent density and female predominance, alongside *Stomoxys* and *Tabanus* species that may contribute to mechanical transmission. Based on evidence from previous studies and broader epidemiological considerations, community‑based vector control, farmer training and awareness, and integration into regional livestock health programs are potential strategies that could strengthen trypanosomosis management in endemic areas. In addition, the development of longitudinal and molecular‑based studies is recommended to refine epidemiological understanding.

## Ethical statement

Ethical approval for animal sampling was obtained from the Woldia University Research Ethics Committee (Approval No. WU-VM-2024/12). All procedures followed institutional and national guidelines for animal welfare. Farmer consent was obtained prior to sampling.

## AI statement

During the preparation of this work the author(s) used Microsoft copilot in order to rewrite the cover letter and manuscript. After using this tool/service, the author(s) reviewed and edited the content as needed and take(s) full responsibility for the content of the published article.

## Funding information

This research received no specific external funding.

## Clinical trial registration

Not applicable.

## Consent for publication

Not applicable.

## Informed consent statement

Not applicable.

## CRediT authorship contribution statement

**Hirut Getnet Tegegn:** Writing – review & editing, Writing – original draft, Validation, Resources, Methodology, Investigation, Formal analysis, Conceptualization. **Assaye Wollelie Fentie:** Writing – review & editing, Writing – original draft, Visualization, Validation, Software, Resources, Project administration, Methodology, Investigation, Formal analysis, Data curation, Conceptualization. **Animaw Andargie Worku:** Writing – review & editing, Writing – original draft, Validation, Data curation. **Assaye Desta Amare:** Writing – review & editing, Writing – original draft, Validation, Data curation. **Teketay Bayleyegn Derso:** Writing – original draft, Validation, Data curation. **Yihenew Getahun Ambaw:** Writing – original draft, Validation, Data curation. **Getachew Tadesse Siyoum:** Writing – review & editing, Writing – original draft, Visualization, Validation, Supervision, Software, Methodology, Formal analysis, Data curation, Conceptualization.

## Declaration of competing interest

The authors declare that they have no competing interests.

## Data Availability

The datasets used and/or analysed during the current study are available from the corresponding author on reasonable request.
